# A Physical Therapy Clinical Framework for Temporomandibular Disorders: Integrating DC/TMD, ICOP and Multidimensional Patient Phenotyping

**DOI:** 10.3390/jpm16100529

**Published:** 2026-10-14

**Authors:** Paolo Bizzarri, Giacomo Asquini, Cecilia Bagnoli, Giulia Pisacane, Anna Manzari, Paolo Bulzacca, Filippo Maselli, Firas Mourad, Marco Pernici, Andrea Giusti, Aldo Scafoglieri, Edoardo Balli

**Affiliations:** 1 Experimental Anatomy Research Group (EXAN), Vrije Universiteit Brussel (VUB), B-1090 Brussels, Belgium; 2 Department of Human Neurosciences, University of Roma “La Sapienza”, 00185 Rome, Italy; 3 Facoltà Dipartimentale di Medicina e Chirurgia, Università Campus Bio-Medico di Roma, 00128 Rome, Italy; 4 Department of Clinical Science and Translational Medicine, University of Rome “Tor Vergata”, 00133 Rome, Italy; 5 Department of Medicine and Health Science “Vincenzo Tiberio”, University of Molise, 86100 Campobasso, Italy; 6 Department of Medicine and Surgery, LUM University, 70010 Casamassima, Italy; 7 Department of Health, LUNEX University of Applied Sciences, 50, Avenue du Parc des Sports, 4671 Differdange, Luxembourg; 8 Luxembourg Health & Sport Sciences Research Institute A.s.b.l., 50, Avenue du Parc des Sports, 4671 Differdange, Luxembourg; 9 Dipartimento di Scienze Mediche, Chirurgiche e Neuroscienze, University of Siena, 53100 Siena, Italy; 10 Department of Medicine and Surgery, University of Perugia, 06132 Perugia, Italy

**Keywords:** temporomandibular disorders, orofacial pain, physiotherapy, DC/TMD, ICOP, bruxism, clinical reasoning, phenotyping, rehabilitation, pain mechanisms

## Abstract

Temporomandibular disorders (TMDs) are clinically heterogeneous, and patients sharing the same diagnosis may differ substantially in pain mechanisms, functional impairments, behavioral contributors, comorbidities, psychosocial burden, prognosis, and treatment response. Although the Diagnostic Criteria for Temporomandibular Disorders (DC/TMD), the International Classification of Orofacial Pain (ICOP), and the International Classification of Headache Disorders (ICHD-3) provide essential diagnostic standards, they do not directly translate diagnosis into individualized physiotherapy management. This Masterclass proposes an evidence-informed clinical framework designed to bridge this gap and support personalized, evidence-based practice. Developed through targeted literature searches, citation tracking, and narrative synthesis of diagnostic standards, clinical practice guidelines, systematic reviews, consensus documents, and key phenotyping studies, the framework retains established diagnoses as clinical anchors while adding a physiotherapy-oriented translational layer. Assessment focuses on clinically relevant and potentially modifiable features, including familiar pain, mandibular mobility and motor control, behavioral load, regional and sleep-related comorbidities, psychosocial factors, prognosis, and pain-mechanism hypotheses. These findings are integrated with patient goals and preferences to identify dominant treatment targets, inform intervention intensity, select appropriate outcomes, and determine when interdisciplinary care is required. Education, therapeutic exercise, selected manual therapy, and behavioral strategies are therefore considered according to explicit clinical objectives rather than diagnostic labels alone. The framework distinguishes evidence-supported components from extrapolated and hypothesis-generating propositions and aims to make physiotherapy management more transparent, reproducible, patient-centered, and testable.

## 1. Introduction

Temporomandibular disorders (TMDs) are among the most common causes of non-odontogenic orofacial pain and include conditions affecting the temporomandibular joint, masticatory muscles and associated structures [1]. Their clinical presentations are heterogeneous: patients may report pain during jaw function, restricted mouth opening, impaired chewing, joint noises, headache, sleep-related complaints, parafunctional behaviors or broader psychosocial burden. This heterogeneity suggests multidimensional management.

Diagnostic Criteria for Temporomandibular Disorders (DC/TMD) [2], the International Classification of Orofacial Pain (ICOP) [3] and the International Classification for Headache Disorders-3 (ICHD-3) [4] provide the diagnostic foundation and a common language for interdisciplinary communication. Over the last three decades, the field has moved from predominantly mechanical and occlusal explanations toward a multidimensional biopsychosocial profiling [5,6]. The Research Diagnostic Criteria for Temporomandibular Disorders (RDC/TMD) [7] introduced a dual-axis structure in which physical diagnoses were integrated with psychosocial assessment. This approach was refined in DC/TMD, which provided standardized procedures for clinical and research settings, especially for pain-related TMD diagnoses [2]. A clinically important contribution of the DC/TMD is the emphasis on reproduction of the patient’s familiar pain during provocation testing. This criterion helps distinguish clinically meaningful pain from incidental tenderness elicited during examination.

The International Classification of Orofacial Pain (ICOP) published in 2019 further included TMD within the broader taxonomy of orofacial pain [3]. This is particularly relevant for clinicians working in rehabilitation, because patients with jaw pain may also present with odontogenic disorders, neuropathic pain, idiopathic orofacial pain, otological symptoms or systemic contributors. Migraine, tension-type headache or other headache disorders may also be part of the clinical presentation, with ICHD-3 as diagnostic reference standard for headache disorders [4].

However, these classification systems do not directly specify which physiotherapy interventions should be selected, how treatment should be sequenced, which clinical features should be prioritized as modifiable targets, or which patients require broader interdisciplinary management [8,9].

A diagnosis such as myalgia, arthralgia, or headache attributed to TMD identifies the clinical condition but does not fully explain why an individual patient remains symptomatic, which impairments are modifiable, which behaviors may perpetuate symptoms, or how clinical management should be individualized [10].

Two patients with the same DC/TMD diagnosis may differ substantially in mandibular mobility, familiar pain reproduction, movement quality, motor control, pain mechanisms, bruxism, sleep quality, headache burden, disability, expectations, distress, comorbid pain conditions, and response to manual therapy or exercise.

Physiotherapy management therefore requires a translational step between diagnostic classification and conservative treatment planning. In the proposed framework, clinical reasoning progresses from diagnostic anchoring to the identification of a physiotherapy phenotype, the definition of treatment targets, the selection of an appropriate intervention intensity and the monitoring of clinical response. Thus, while the diagnostic label remains necessary, physiotherapy treatment selection is primarily guided by modifiable impairments, pain mechanisms, behavioral load, regional and general comorbidities, psychosocial context, and prognosis. Recent survey data also indicate variability in physiotherapists’ knowledge and clinical management of TMD, further supporting the need for standardized, evidence-informed clinical reasoning frameworks [11].

The purpose of this article is to propose a physiotherapy-oriented clinical framework for the assessment and management of TMD.

The present framework was developed primarily for adult patients because most of the diagnostic validation and treatment literature informing its development derives from adult populations. Children and adolescents require age-appropriate assessment of symptoms, development, psychosocial factors and functional impact. Specific adaptations of the DC/TMD have been developed for children and adolescents, supporting the need for developmentally appropriate diagnostic procedures. Therefore, several principles of the present framework may be conceptually transferable to younger populations, but its direct application to children and adolescents should not be assumed without age-specific adaptation and validation [12].

The framework integrates DC/TMD, ICOP and ICHD-3-informed differential diagnosis with direct-access screening, standardized clinical examination, behavioral assessment, pain-mechanism-informed reasoning, prognostic profiling and evidence-informed physiotherapy interventions. It is not intended as a formal clinical practice guideline or a validated prediction model, but as a structured, evidence-informed clinical reasoning model to support individualized treatment planning, improve transparency in decision-making and generate testable hypotheses for future research.

## 2. Development of the Framework

This Masterclass was developed as an evidence-informed expert synthesis. The manuscript is not intended to provide a systematic review of the literature, but rather to offer a critical and practice-oriented interpretation of current knowledge relevant for musculoskeletal physiotherapy. The relevant literature was identified through targeted database searches and citation tracking. Evidence was synthesized narratively, and expert opinion (P.Bi., G.A., C.B., G.P., A.M., P.Bu., F.Ma, F.Mo., M.P., A.G., A.S., and E.B.) was used to contextualize the findings, establish clinical relevance, and identify future research directions.

Targeted searches were conducted in MEDLINE, Scopus, Web of Science and Embase from September 2025 to February 2026, supplemented by backward and forward citation tracking of key documents. Search concepts included temporomandibular disorders, orofacial pain, DC/TMD, physiotherapy, rehabilitation, manual therapy, therapeutic exercise, bruxism, pain mechanisms, phenotyping, prognosis, sleep and headache. Evidence was prioritized according to clinical relevance and hierarchy of source, with international diagnostic standards and consensus documents used to define diagnostic constructs. TMD-specific clinical practice guidelines and systematic reviews informed management, observational and phenotyping studies informed prognosis and multidimensional characterization, and the broader musculoskeletal literature was used only where TMD-specific evidence was unavailable. No formal risk-of-bias assessment, systematic study selection or GRADE evaluation was undertaken because the objective was conceptual framework development rather than systematic evidence synthesis.

The literature base prioritized: (1) DC/TMD and related diagnostic documents [2,13]; (2) ICOP and ICHD-3 criteria relevant to orofacial pain and headache attributed to TMD [3,4]; (3) recent clinical practice guidelines and systematic reviews on conservative treatment for TMD-associated pain [14,15]; (4) international consensus documents on bruxism and ecological momentary assessment [16,17,18]; (5) cohort and phenotyping studies, including OPPERA, that support the clinical relevance of psychosocial burden, pain sensitivity and comorbid symptoms [8,19]; and (6) physiotherapy-oriented classification and stratified-care models developed in other musculoskeletal fields, including movement-system impairment frameworks, clinical practice guidelines, motor-control classifications and prognostic stratification models [20,21].

The framework is intended to inform clinical reasoning across both recent-onset and persistent/chronic TMD presentations. Pain duration was therefore considered as a clinical and prognostic modifier rather than as a separate phenotype. However, the evidence base is not equivalent across stages of pain persistence, as several contemporary guidelines and evidence syntheses informing treatment recommendations specifically address chronic painful TMD. Accordingly, treatment propositions for recent-onset presentations should be interpreted more cautiously where direct TMD-specific evidence is limited.

The framework was constructed through clinical synthesis rather than formal evidence grading. Therefore, recommendations are expressed as clinical reasoning propositions. To avoid conflating established evidence with proposed clinical reasoning, the framework distinguishes three levels of evidential support: (1) components directly supported by TMD-specific diagnostic standards, clinical practice guidelines or systematic reviews; (2) components informed by evidence extrapolated from the broader musculoskeletal or pain literature; and (3) hypothesis-generating propositions derived from integration of available evidence and expert clinical reasoning. Components in the third category are intended to generate testable treatment hypotheses and should not be interpreted as validated treatment-selection rules.

This article applies a similar logic from treatment-based and stratified-care principles used in musculoskeletal physiotherapy to TMD care: the diagnosis anchors the clinical condition, but physiotherapy phenotyping identifies potential modifiable targets and may inform treatment intensity, monitoring and referral, integration of modifiable impairments, movement deficits, motor-control disturbances, pain mechanisms, psychological risk, functional limitation and expected prognosis.

## 3. Core Premise: Biomedical Diagnosis Is Necessary but Not Sufficient

**Figure 1:**
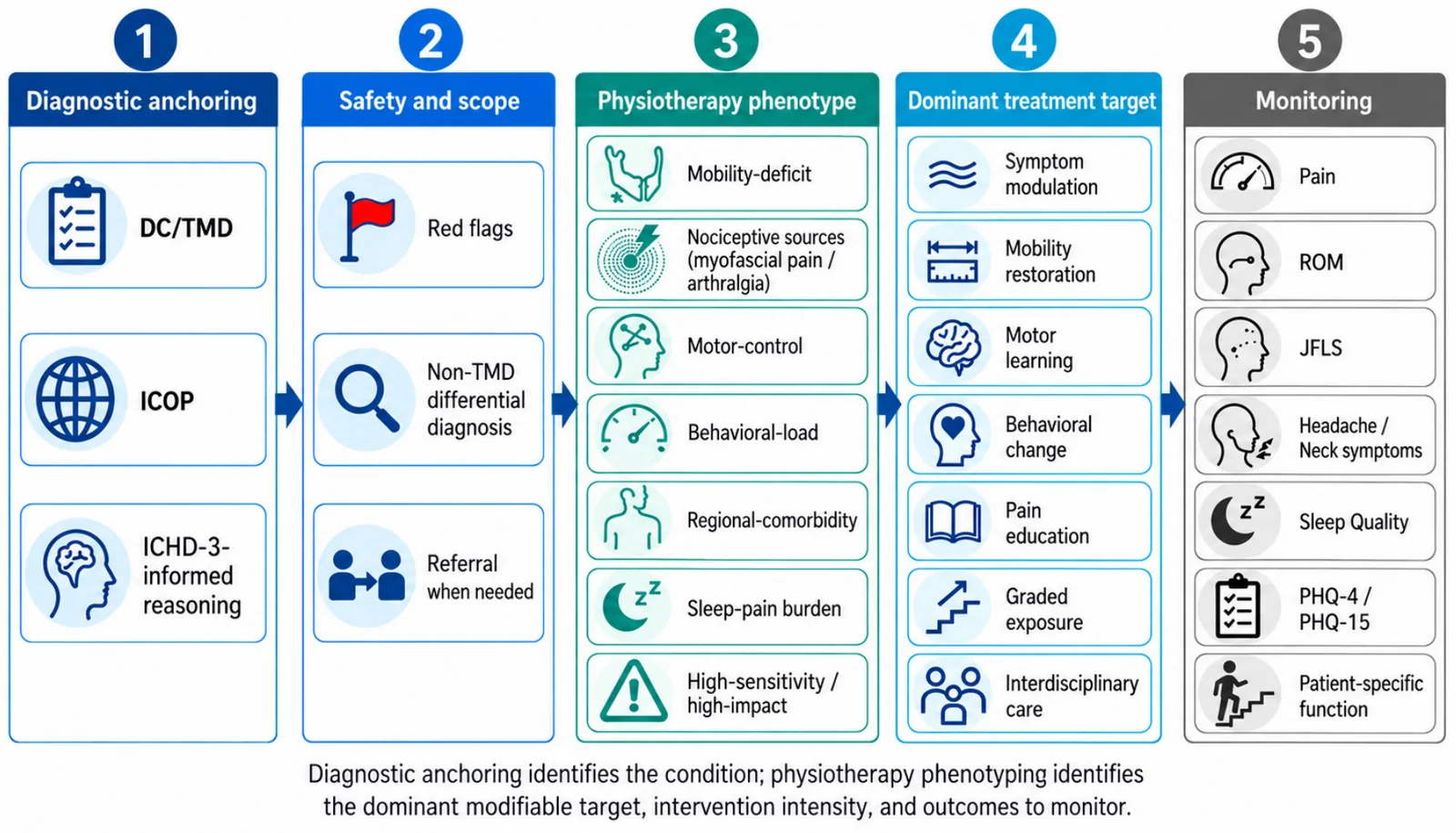
Clinical reasoning pathway in physiotherapy-oriented TMD management.

TMD diagnosis and physiotherapy treatment selection are related but not equivalent tasks. Biomedical diagnosis classifies the disorder; physiotherapy phenotyping identifies the clinically relevant and potentially modifiable contributors to pain and disability (Figure 1).

DC/TMD, ICOP and ICHD-3 answer questions such as the following: Is the patient’s pain compatible with migraine, tension-type headache or another orofacial pain condition? Are specific painful and/or intra-articular TMD diagnoses supported or excluded according to established diagnostic criteria? Physiotherapy assessment should then answer additional questions: Do other findings not included in the standard DC/TMD examination, such as assessment of the hyoid muscles, reproduce the patient’s familiar pain? Is mandibular movement limited, poorly coordinated or fearfully guarded? Are joint sounds incidental or functionally relevant? Is awake bruxism present as a potential perpetuating behavior? Are symptoms predominantly local and load-related, show features raising suspicion of neuropathic pain, or embedded within a broader high-sensitivity presentation? Is sleep disturbance, regional comorbidity or psychosocial burden likely to influence prognosis?

This distinction has practical implications. A patient with local myalgia, low disability, normal psychosocial screening and clear reproduction of familiar pain during masseter palpation may respond to education, load management, targeted exercise and short-term symptom-modulating manual therapy [22]. In contrast, a patient with the same diagnostic label but high disability, frequent migraine, widespread tenderness, poor sleep, severe distress and multiple comorbid symptoms may require a broader and more interdisciplinary approach [8]. The diagnostic label is the same, the physiotherapy pathway is not.

Therefore, the framework should be understood as a physiotherapy-oriented translation model. Diagnostic anchoring identifies the condition; physiotherapy phenotyping identifies the dominant modifiable target, the appropriate treatment intensity and the outcomes that should be monitored.

## 4. Clinical Reasoning Pathway: From Diagnostic Anchoring to Outcome Monitoring

In direct-access physiotherapy settings, the first clinical question is not which treatment should be applied, but whether the patient can be safely and appropriately managed within the physiotherapist’s scope of practice [23]. For this reason, the proposed framework starts with a sequential clinical reasoning pathway that combines screening for serious conditions (red flags), screening for specific conditions requiring referral to a different specialist, diagnostic anchoring, physiotherapy phenotyping, treatment-target selection, care-intensity matching and outcome monitoring.

The pathway is summarized in Figure 1. For conceptual clarity, Figure 1 presents diagnostic anchoring as the entry point to the framework, followed by safety and scope assessment. In clinical practice, however, safety screening is not restricted to a single sequential step and should be considered from first contact and revisited whenever new clinical information emerges. When severe pathology or a condition outside the physiotherapist’s scope of practice is suspected, the appropriate management decision is referral rather than physiotherapy treatment [23]. This includes, for example, suspected neurological disease, infection, malignancy, acute trauma, progressive systemic disease, or other presentations requiring medical or dental diagnosis before conservative rehabilitation [24] (Box 1).

Box 1 **Red flags and referral considerations.**

Physiotherapists managing TMD should consider referral or interdisciplinary consultation when patients present with

- Progressive neurological deficits;
- Electric-shock-like paroxysmal pain suggestive of trigeminal neuralgia;
- Unexplained weight loss or systemic illness;
- Fever;
- Infection signs or acute swelling;
- History of malignancy;
- Recent trauma with suspected fracture or dislocation;
- Inflammatory polyarthritis;
- Rapidly progressive occlusal change;
- Persistent unilateral otological symptoms not explained by TMD;
- Severe sleep-disordered breathing symptoms;
- Severe psychological distress and high-impact chronic pain requiring multidisciplinary care.

The second decision level is screening for specific conditions that may influence management or require co-management. Rheumatological conditions, clinically relevant sleep bruxism [25], suspected sleep-disordered breathing [26], complex headache presentations or other systemic contributors should not necessarily exclude physiotherapy, but they may require interdisciplinary management [27]. In this context, physiotherapy can remain part of the care pathway, provided that the relevant medical, dental, sleep-medicine, psychological, neurological or orofacial pain expertise is involved when appropriate.

Once referral needs and specific conditions have been considered, diagnostic anchoring is used to identify pain-related and intra-articular TMD presentations and to consider competing or coexisting diagnoses. In the proposed framework, the diagnostic label is then translated into a physiotherapy phenotype. This includes assessment of mobility deficits, familiar pain reproduction, movement quality, motor control, behavioral load, pain mechanisms, regional comorbidities, sleep burden and psychosocial complexity (yellow flags). These findings are interpreted according to their clinical relevance, association with familiar symptoms, functional impact and modifiability.

The final step is not simply selecting an intervention but defining a dominant treatment target and using clinical complexity to inform intervention intensity. A patient may primarily require pain modulation, mobility improvement, motor learning, behavioral load reduction, pain education, graded exposure or interdisciplinary care. Treatment response should then be monitored using outcomes aligned with the dominant phenotype.

Overall, diagnostic anchoring identifies the condition, whereas physiotherapy phenotyping identifies the dominant modifiable target, the appropriate intensity of intervention, and the most relevant outcomes to monitor.

## 5. Domain 1: Diagnostic Anchoring and Differential Diagnosis

The first clinical domain of the framework is diagnostic anchoring. Physiotherapy assessment should begin from, or be integrated with, a recognized and validated diagnostic system, following international consensus and established reference standards. Establishing whether the presentation is compatible with a TMD is important for identifying a musculoskeletal orofacial condition and defining the boundaries of physiotherapy management. DC/TMD provides standardized procedures for the most common TMD diagnoses and is particularly robust for pain-related conditions [2]. ICOP supports broader differential diagnosis in orofacial pain and helps situate TMD within a larger clinical taxonomy [3]. ICHD-3 is relevant when headache attributed to TMD coexists with, or must be differentiated from, primary headache disorders such as migraine or tension-type headache and secondary headache disorders such as cervicogenic headache.

A physiotherapy-oriented framework should not encourage diagnostic isolation. Jaw pain is not always TMD, and marked jaw functional limitations may coexist with little or no pain. Physiotherapists should screen for features suggesting odontogenic pain, trigeminal neuralgia or other neuropathic pain, inflammatory arthropathy, trauma-related pathology, malignancy, or primary headache disorders requiring medical management. This is particularly important because some clinical findings, such as joint sounds, tenderness or limited opening, may occur in more than one condition and are not automatically treatment targets [13,28].

Within this framework, diagnostic anchoring has several functions. It prevents over-treatment of incidental findings; provides a common language for communication with dentists, neurologists, physicians, psychologists and other clinical professionals; defines the boundaries of physiotherapy care; and identifies cases of patients with orofacial pain requiring referral or co-management [29].

**Figure 2:**
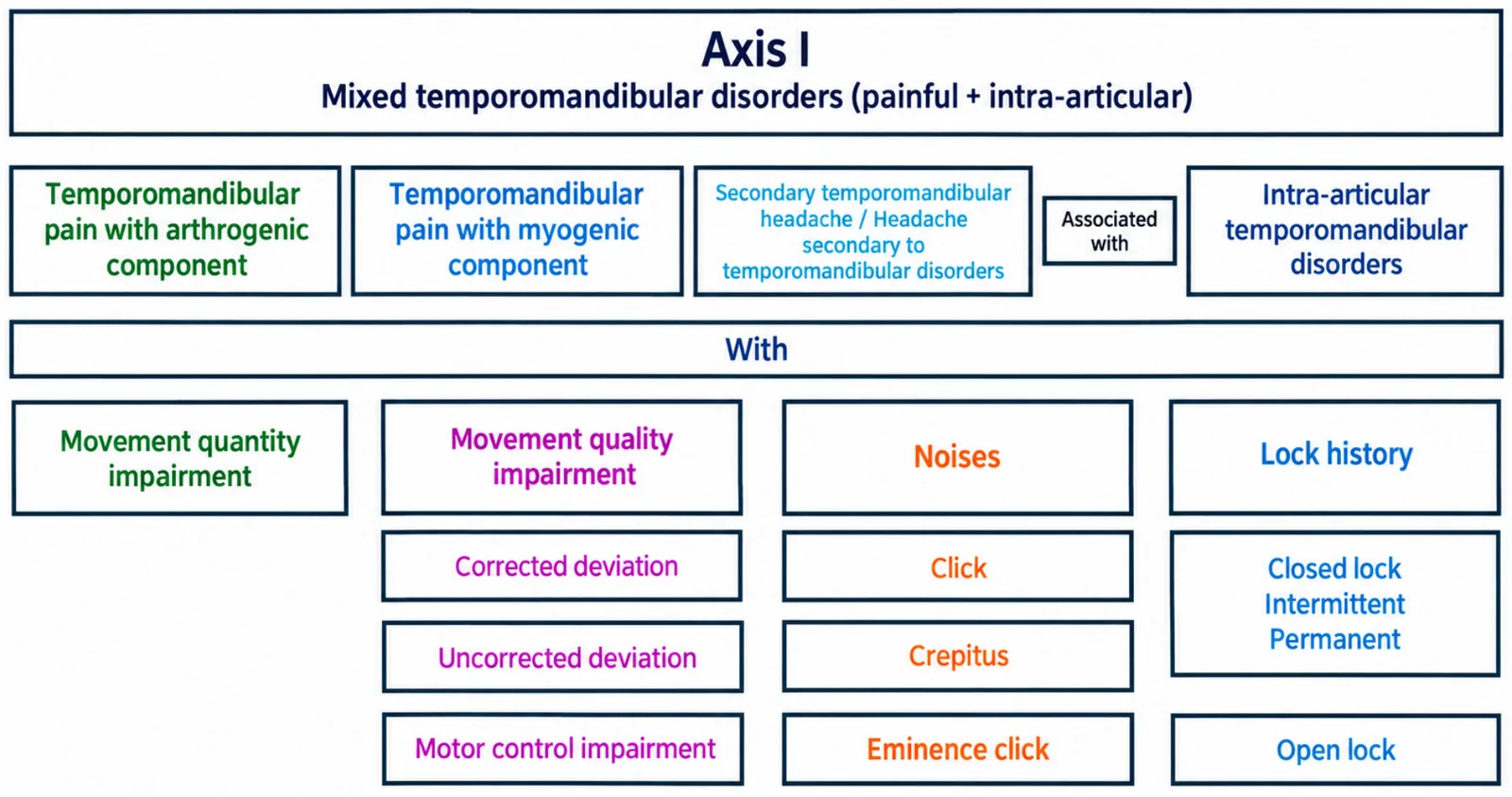
Proposed physiotherapy-oriented translation of Axis I findings in mixed temporomandibular disorder presentations. Mixed TMD is used within the proposed framework as a pragmatic descriptor for the coexistence of pain-related and intra-articular TMD diagnoses. The associated physiotherapy assessment considers these findings as potential treatment targets only when they are associated with familiar symptoms, functional limitation, avoidance behavior, or meaningful patient concern.

Within the proposed framework, Axis I diagnostic findings can be translated into physiotherapy-relevant assessment domains without altering the original DC/TMD classification (Box 2). A mixed TMD presentation is used here as a pragmatic descriptor for the coexistence of one or more pain-related TMD diagnoses, such as myalgia, arthralgia, or headache attributed to TMD, with an intra-articular TMD diagnosis. Physiotherapy assessment should then determine whether this combination is associated with clinically relevant alterations in movement quantity, quality, motor control, symptomatic joint noises, or a history of locking (Figure 2). These findings should not be considered automatic treatment targets. Their relevance depends on their association with familiar symptoms, functional limitation, avoidance behavior, or meaningful patient concern.

Box 2 **What this framework is and what it is not.**

This framework is:

- A physiotherapy-oriented translation layer between diagnosis and treatment;
- A structured clinical reasoning model;
- A way to connect diagnosis with modifiable treatment targets;
- A basis for individualized conservative care and stepped interdisciplinary management;
- A proposal requiring feasibility, reliability, prognostic and clinical validation.

This framework is not:

- A replacement for DC/TMD, ICOP or ICHD-3;
- A new diagnostic classification of TMD;
- A protocol assigning the same treatment to all patients with the same diagnosis;
- A claim that all TMD symptoms are driven by movement impairment;
- A claim that physiotherapists should manage complex headache, sleep or psychological disorders independently.

## 6. Domain 2: Peripheral Nociceptive Pain Contributors and Familiar Pain

The second domain is the identification of peripheral nociceptive contributors that are clinically meaningful for the individual patient. The central concept is familiar pain. Pain provoked during examination should not be interpreted solely according to intensity or anatomical location. It should be interpreted according to whether the patient recognizes it as similar to the pain experienced in daily life.

In DC/TMD, familiar pain is central to the diagnosis of pain-related TMD [2]. Within the proposed framework, familiar pain reproduction may also inform the identification of clinically relevant treatment targets. If palpation of the masseter or temporalis reproduces familiar pain, this finding supports a possible myogenic contribution and informs a treatment hypothesis involving education, exercise, self-management and selected manual therapy. If joint loading or joint play reproduces familiar pain, an articular contribution may be clinically relevant. If examination findings are painful but unfamiliar, they should be interpreted more cautiously, especially in patients with high generalized sensitivity.

Standardized palpation improves interpretability. For primary diagnostic sites, DC/TMD-based palpation of the masseter and temporalis is performed with standardized pressure and duration. The assessment should distinguish local myalgia, myofascial pain and myofascial pain with referral. However, the framework also recognizes that physiotherapy examination may extend beyond diagnostic palpation to include assessment of movement, function, load tolerance and symptom modification. Supplementary palpation sites, intra-oral examination or temporalis tendon assessment may refine clinical hypotheses, but their diagnostic weight should be lower than that of standardized DC/TMD procedures unless reliability and clinical relevance are established.

Joint assessment, including joint accessory movements, should follow the same logic. Imaging findings, joint noises or deviation in mouth opening should not automatically be considered pathological. Disc displacement with reduction and joint sounds are common and may be present without pain or disability. The physiotherapy question is not whether a joint sound exists, but whether it is associated with familiar pain, functional limitation, locking, avoidance behavior or meaningful patient concern. Similarly, manual assessment of joint mobility may inform treatment planning when it identifies relevant limitation, symptom modification, familiar pain reproduction or clear functional impairment.

### Pain-Mechanism-Informed Phenotyping

**Figure 3:**
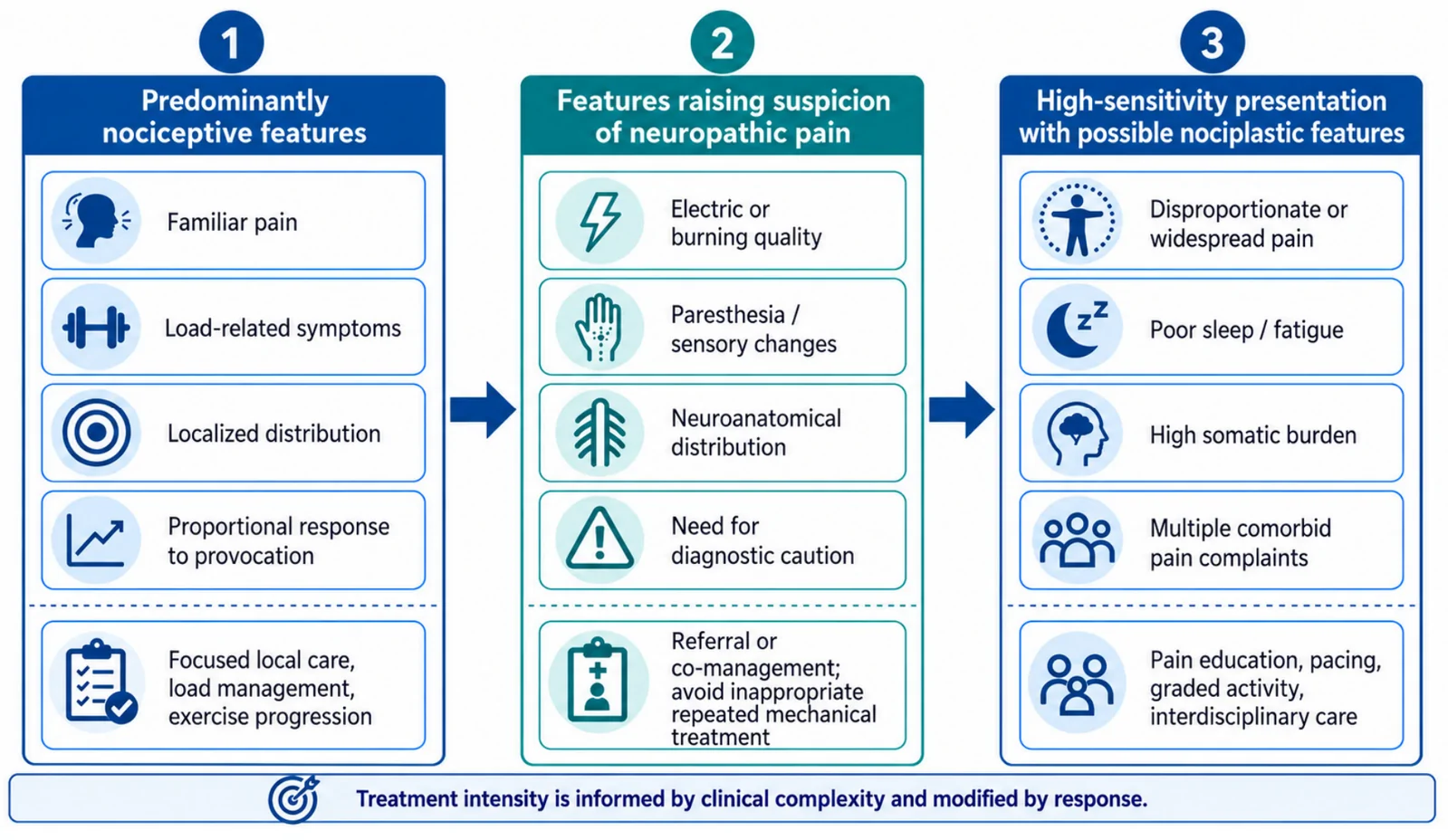
Pain-mechanism-informed phenotyping in TMD.

The terminology used here describes clinical features that may inform mechanistic hypotheses rather than establish mechanism-based pain diagnoses (Table 1). In particular, symptoms such as burning pain, allodynia or paresthesia may raise suspicion of neuropathic pain but do not establish it in the absence of evidence of a lesion or disease affecting the somatosensory nervous system. Similarly, widespread pain, fatigue or high somatic burden may suggest a high-sensitivity presentation but are not sufficient to establish an IASP-defined nociplastic pain diagnosis. Nociceptive, neuropathic and nociplastic mechanisms may also coexist within the same patient (Figure 3).

Familiar pain and local symptom reproduction help identify potentially relevant peripheral nociceptive contributors, but physiotherapy reasoning should also consider the dominant pain mechanism. Pain-mechanism-informed phenotyping has been proposed in musculoskeletal physiotherapy to distinguish clinical presentations with predominantly nociceptive features, features raising suspicion of neuropathic pain, or a high-sensitivity presentation with possible nociplastic features [20,21]. In the present framework, this distinction is not used as a rigid diagnosis, but as a clinical reasoning aid that may inform treatment-target hypotheses, referral decisions and expectations regarding clinical response.

Predominantly nociceptive features are suggested when pain is anatomically plausible, related to mechanical load or jaw function, proportionate to provocation, and clearly reproduced as familiar pain during examination. Within the proposed framework, such presentations may support focused conservative care, including education, load management, mobility or strengthening exercises, behavioral modification, and selected manual therapy [30].

Features raising suspicion of neuropathic pain require a different interpretation. Electric-shock-like pain, burning quality, paresthesia, sensory loss, allodynia in a neuroanatomically plausible distribution, cranial nerve signs or paroxysmal attacks not consistent with musculoskeletal behavior should prompt diagnostic caution and referral or co-management. In these cases, repeated local mechanical treatment may be inappropriate unless a clear musculoskeletal contributor is also present [30].

A high-sensitivity presentation with possible nociplastic features may be considered when pain is disproportionate to local findings; widespread; and associated with generalized hypersensitivity, poor sleep, fatigue, high somatic symptom burden, distress or multiple comorbid pain complaints. These patients may still benefit from physiotherapy, but the proposed treatment emphasis may include education, graded activity, functional restoration and interdisciplinary care rather than repeated local symptom provocation or passive treatment escalation.

## 7. Domain 3: Mandibular Function, Movement Quality and Motor Control

The third domain concerns mandibular function, movement quality and motor control. TMD management should not be limited to pain provocation. Functional assessment should include mouth opening, lateral excursions, protrusion, chewing and speech-related symptoms, masticatory muscle fatigue, avoidance behaviors, and patient-specific functional limitations.

Maximum mouth opening is clinically useful but insufficient alone. Two patients may have the same opening range but differ substantially in pain, movement smoothness, deviation, confidence and functional capacity. Therefore, clinicians should observe the quality of opening and closing, the presence of deflection or deviation, the ability to perform slow controlled movement, the effect of visual feedback, and the relationship between movement and familiar symptoms [31].

In physiotherapy, the temporomandibular region should be considered part of the broader movement system. Jaw opening, cervical control and functional tasks such as chewing, speaking and yawning may interact within the patient’s symptom behavior. Therefore, the clinical target is not the temporomandibular joint in isolation, but the modifiable movement strategy associated with pain, disability or functional limitation.

Motor control assessment is particularly relevant when patients show aberrant movements, excessive guarding, asymmetrical opening, repeated checking behaviors or difficulty relaxing the jaw at rest [32]. In such cases, the treatment target is not merely increasing range of motion but improving movement confidence, coordination and load tolerance. Exercises may include graded exposure to feared movements, mirror feedback, gentle isometric contractions, resisted lateral movements, relaxation of unnecessary jaw muscle activity and task-specific functional training [33].

Exercise prescription should be objective-driven. Stretching and mobility exercises may be considered when limited range of motion or functional restriction is present. Coordination exercises may be considered when movement quality is impaired. Isometric or resisted exercises may be used when pain modulation, load tolerance or confidence is the target. Habit-reversal and rest-position training may be particularly relevant when awake bruxism or jaw bracing are prominent. This individualized approach is intended to avoid prescribing the same exercise package to all TMD patients regardless of phenotype [34,35,36].

## 8. Domain 4: Behavioral and Lifestyle-Related Perpetuating Factors

The fourth domain addresses behavioral and lifestyle-related factors that may perpetuate symptoms. Among these, awake bruxism is particularly relevant for physiotherapy because it may involve sustained tooth contact, clenching, jaw bracing or other non-functional masticatory muscle activities during wakefulness [17].

Contemporary consensus definitions emphasize that bruxism should not be automatically conceptualized as a disorder [37]. It is better understood as a spectrum of masticatory muscle activities that may be harmless in some individuals and clinically relevant in others. The clinical question is therefore not simply whether bruxism is present, but whether specific behaviors contribute to pain, fatigue, disability, muscle overload or patient distress. Recent evidence also suggests a dose–response relationship between waking-state oral behaviors and psychological distress, reinforcing the need to interpret awake bruxism within the broader behavioral and psychosocial context [38].

Awake bruxism and sleep bruxism should be distinguished. Sleep bruxism is a centrally regulated sleep-related masticatory muscle activity and should not be inferred from daytime clenching alone [39]. Awake bruxism is more accessible to behavioral modification because it occurs during consciousness and can be monitored in daily life. Self-reported questionnaires are useful but vulnerable to recall bias and distress-related overestimation. Ecological momentary assessment, including smartphone-based approaches, provides real-time sampling of jaw behaviors in the patient’s natural environment and may improve awareness of patterns such as jaw bracing, light tooth contact or clenching [40].

Within the proposed framework, ecological momentary assessment is used primarily as an assessment and awareness tool. Ecological momentary intervention may then be used to prompt jaw relaxation, mandibular rest-position training or behavior interruption in real-life contexts. This is a promising strategy, but its clinical superiority over standard counseling should not be assumed without further comparative evidence. It should be presented as an evidence-informed behavioral extension rather than as a stand-alone proven treatment.

Other lifestyle-related factors may also influence prognosis and treatment tolerance. Sleep disturbance, high stress, low physical activity, and broader somatic symptom burden can influence response to local treatment [41]. Physiotherapists should screen for these factors and consider education, sleep hygiene advice and referral when indicated. However, these elements should be individualized and kept within the professional scope of practice.

## 9. Domain 5: Prognostic Profiling, Comorbidities and Stratified Stepped Care

**Figure 4:**
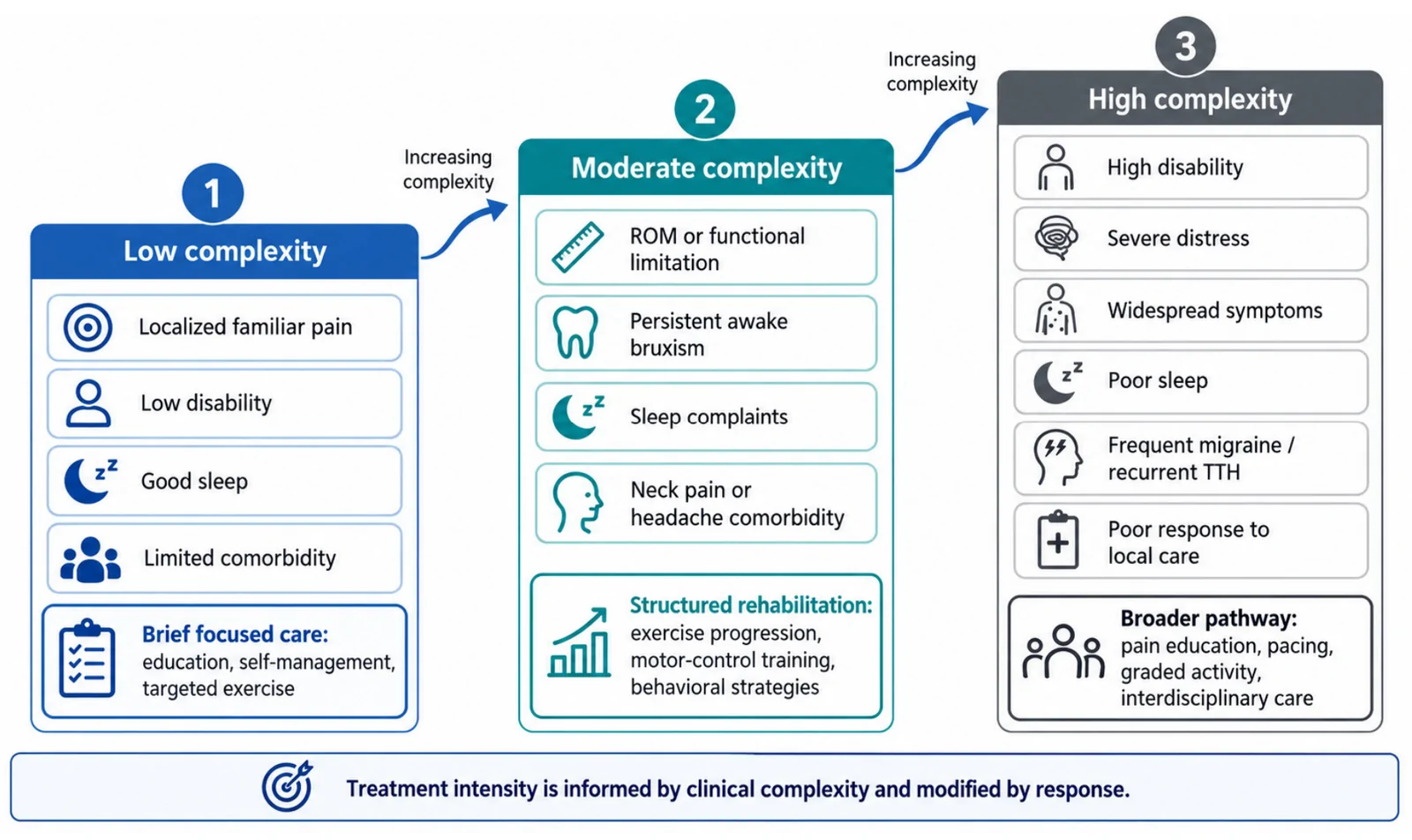
Proposed stratified stepped-care model in physiotherapy-oriented TMD management.

The fifth domain is prognostic profiling. In TMD, prognosis is influenced not only by local tissue findings but also by pain intensity, pain duration, disability, distress, sleep quality, regional comorbidities and widespread pain sensitivity (Figure 4). This is consistent with the broader biopsychosocial model of persistent pain and with cohort data showing that multidimensional phenotypes are clinically relevant [19,42].

Axis II assessment should therefore represent a core component of multidimensional physiotherapy assessment. Instruments such as the Graded Chronic Pain Scale, PHQ-4, PHQ-9, GAD-7, PHQ-15, Jaw Functional Limitation Scale and pain drawings can help quantify pain impact, distress, somatic symptoms and functional limitation [43]. Meta-analyses also indicate that depression is differentially associated with TMD diagnostic groups, particularly pain-related TMD, supporting the clinical relevance of psychosocial profiling [44]. These tools do not replace clinical judgment, but they make risk stratification more transparent and may help inform whether care remains locally focused or whether broader interdisciplinary management should be considered.

Regional comorbidities are clinically important because TMD symptoms often occur together with neck pain [45], migraine, tension-type headache [46] or mixed craniofacial pain complaints [47]. These conditions can influence pain intensity, disability, prognosis and treatment response. Within this framework, they should not be interpreted as automatic causes of TMD, but as interacting factors that refine clinical reasoning. Neck pain may coexist with TMD through shared functional and neurophysiological mechanisms involving the trigeminal and upper cervical systems. Clinically, physiotherapists should assess whether neck-related findings reproduce familiar symptoms, modify jaw or headache complaints, or contribute to functional limitation. The presence of neck pain alone does not imply that the cervical region is the primary driver.

Migraine and tension-type headache also require careful interpretation. TMD pain may coexist with primary headache disorders, contribute to headache-like symptoms, or be part of headache attributed to TMD [46]. ICOP- and ICHD-3-informed reasoning is therefore useful to distinguish comorbidity from causal attribution. Frequent headache, medication use, sensory sensitivity and high pain-disability should increase clinical vigilance and may indicate the need for interdisciplinary management. Localized TMD with mild neck or headache symptoms may remain within a focused conservative pathway, whereas frequent migraine, recurrent tension-type headache, high disability or widespread regional symptoms may require broader rehabilitation and medical, neurological or orofacial pain collaboration.

Sleep is another relevant prognostic and treatment-modifying domain. Poor sleep can amplify pain, reduce recovery and worsen disability [42]. Sleep disturbance may be both a consequence of persistent orofacial pain and a contributor to symptom persistence [48]. Clinicians should distinguish poor sleep quality, insomnia-like symptoms, non-restorative sleep, morning jaw pain or fatigue, suspected sleep bruxism, and symptoms suggestive of sleep-disordered breathing. These presentations have different implications. Poor sleep quality may indicate reduced recovery capacity and increased pain sensitivity, whereas loud snoring, witnessed apneas or excessive daytime sleepiness should suggest referral for sleep-medicine assessment.

Within this framework, sleep is considered a prognostic and treatment-modifying factor. A patient with localized familiar pain, low disability and good sleep may respond to focused conservative care, while a patient with persistent insomnia, fatigue, headache comorbidity or high symptom burden may justify consideration of a slower, broader and more supported pathway. Physiotherapy management may include education on the sleep–pain relationship, relaxation strategies and basic sleep hygiene advice. However, physiotherapists should avoid managing complex insomnia or suspected sleep-disordered breathing independently.

Pain duration should contribute to prognostic profiling, since a recent-onset, localized presentation and a persistent pain presentation with disability, sleep disturbance or multiple comorbidities may require a different treatment intensity despite sharing the same DC/TMD diagnosis.

The framework proposes a pragmatic stratified stepped-care model organized according to clinical complexity, treatment intensity and criteria for escalation. This structure is inspired by stratified-care approaches used in musculoskeletal physiotherapy, where treatment intensity is matched to clinical risk rather than applied uniformly to all patients [49].

OPPERA and related phenotyping studies support the clinical relevance of multidimensional profiles, including adaptive, pain-sensitive and global-symptom patterns [19]. However, these clusters should inform clinical reasoning rather than be applied as discrete categories. The framework therefore uses prognostic profiling, regional comorbidities and sleep assessment as guides to treatment intensity and referral, not as deterministic classification systems (Table 2).

## 10. From Phenotype to Intervention: An Objective-Driven Treatment Sequence

The proposed intervention model follows an objective-driven sequence. Treatment begins with patient education and shared explanation of the clinical phenotype. The patient should be educated on which findings are likely relevant, which findings are incidental, what can be modified and what outcomes will be monitored. This is particularly important for patients concerned about joint noises or imaging findings, because reassurance and explanation may reduce fear-driven monitoring and unnecessary treatment seeking [50].

The same intervention may serve different purposes across phenotypes. Manual therapy may be used for short-term symptom modulation in a local nociceptive presentation, but it should not be the primary strategy in a high-sensitivity or high-impact phenotype [51]. Exercise may target mobility, motor control, graded exposure, load tolerance or self-efficacy depending on the dominant clinical presentation. Education may provide simple reassurance in a low-complexity phenotype, but it may need to address pain mechanisms, recovery, and threat reduction in a high-complexity phenotype [52]. Therefore, interventions are not presented as generic modalities but as tools that may be selected in relation to explicit treatment targets.

Selection of the dominant treatment target should be performed through shared decision-making, integrating the clinician’s assessment of modifiable contributors with the patient’s goals, preferences, expectations and treatment priorities.

### 10.1. Education and Self-Management

Education is the foundation of care. It should include information on the benign and fluctuating nature of many TMD presentations, the limited meaning of isolated joint sounds, the role of load management, the importance of avoiding excessive checking behaviors, and the rationale for active rehabilitation [53,54]. Education should be individualized. A patient with localized pain and low distress may need simple reassurance and practical advice. A patient with persistent pain, high threat appraisal, poor sleep or widespread symptoms may require a broader pain neuroscience-informed explanation, emphasizing sensitivity, recovery, graded exposure and self-efficacy.

### 10.2. Manual Therapy as a Selected Adjunct

Manual therapy should be framed as a selected adjunct rather than a default treatment [22,52,55]. Articular techniques may be considered when joint assessment identifies familiar pain, relevant mobility limitation, movement restriction or functional impairment. Soft-tissue techniques may be considered when palpation reproduces familiar myogenic pain and the intervention improves symptoms, movement or confidence. Manual therapy should not be justified by the intention to recapture a disc, eliminate an asymptomatic click or normalize incidental structural findings [56].

The expected role of manual therapy is symptom modulation, improvement of short-term mobility, facilitation of exercise and reduction of threat [57]. It should generally be combined with active strategies rather than used as an isolated passive treatment. Treatment response should be assessed using patient-specific functional outcomes, pain during relevant tasks, maximum mouth opening when limited, jaw function and self-management confidence. In high-sensitivity or high-impact presentations, manual therapy should be used cautiously and should not replace education, graded activity and interdisciplinary management [7,22].

### 10.3. Therapeutic Exercise and Motor Learning

Therapeutic exercise may be selected according to the dominant functional target. Mobility exercises may be prioritized when range limitation is clinically relevant. Coordination exercises may be considered when movement quality is impaired. Isometric or resisted exercises may be used for pain modulation, load tolerance and confidence. Functional exercises may be introduced when symptoms are task-specific, such as chewing, yawning, speaking or prolonged mouth opening [35].

Exercise should be progressed gradually and monitored according to symptom response. The goal is not simply to increase repetitions, but to restore confident, efficient and non-threatening mandibular function. In patients with high sensitivity or fear, exercise may need to be framed as graded exposure rather than mechanical correction. In patients with regional comorbidities, exercise may also need to consider cervical load tolerance, headache behavior, fatigue and sleep-related recovery.

### 10.4. Behavioral Strategies for Awake Bruxism

When awake bruxism is clinically relevant, the treatment target is awareness and a reduction in sustained non-functional jaw muscle activity [13,58,59]. Patients can be taught a simple mandibular rest position: lips together or relaxed, teeth apart, tongue resting gently on the palate or floor of the mouth according to comfort, and jaw muscles relaxed. However, the instruction should avoid excessive self-monitoring, which may increase vigilance in some patients.

Ecological momentary assessment may be used to identify daily patterns [58]. Ecological momentary intervention may then prompt brief relaxation, breathing, jaw release or task interruption in high-risk contexts. Behavioral strategies should be presented as experiments and adjusted according to patient response. In patients with high distress, awake bruxism may be one expression of broader arousal or coping patterns; in these cases, physiotherapy alone may be insufficient and psychological support may be appropriate.

### 10.5. Interdisciplinary Care and Referral

Collaboration with speech-language therapists may also be appropriate when TMD is accompanied by clinically relevant alterations in chewing, swallowing, speech or broader orofacial myofunction. Orofacial myofunctional therapy may include functional exercises and behavioral strategies directed toward coordinated orofacial activity [59]. However, evidence specifically supporting orofacial myofunctional therapy for TMD remains limited and heterogeneous.

The framework supports conservative physiotherapy but also emphasizes boundaries of care. Patients with suspected neuropathic pain, primary headache requiring medical management, significant sleep-disordered breathing, inflammatory disease, severe psychological distress, persistent high-impact pain or non-response to appropriate conservative care should be considered for interdisciplinary assessment. Physiotherapists should not attempt to manage all drivers independently.

## 11. Operationalizing Physiotherapy Phenotypes: Clinical Tools, Shared Decision-Making and Outcome Monitoring

The framework can be operationalized through physiotherapy-relevant potential phenotypes. Phenotype identification should not be interpreted as an algorithm that automatically assigns a specific intervention. These phenotypes are not mutually exclusive and should not be interpreted as fixed categories. Rather, they help clinicians identify the dominant modifiable target at a given stage of care. A patient may shift from one dominant phenotype to another as symptoms improve, behavior changes or new clinical information emerges.

### 11.1. From Phenotype Identification to Patient-Prioritized Treatment Targets

The dominant treatment target should emerge from integration of clinical findings with the patient’s goals, preferences, expectations, treatment priorities and individual context. Shared decision-making is therefore an essential component of the proposed framework. Clinician-identified modifiable contributors may not necessarily represent the outcomes that matter most to the patient; therefore, treatment planning should prioritize areas in which clinical relevance and patient-prioritized functional goals converge.

Patient-specific goals should be defined at baseline and revisited throughout care. For example, restoration of comfortable chewing, yawning, speaking, prolonged mouth opening, work-related activities or participation in social activities may be more meaningful than isolated changes in pain intensity or range of motion. This approach positions physiotherapy phenotyping within personalized and value-based rehabilitation rather than as a clinician-driven classification system.

### 11.2. Clinical Tools and Monitoring

Assessment tools should be selected according to the clinical question rather than administered uniformly to every patient. Within the proposed framework, a minimum assessment should document diagnosis, pain and familiar symptom reproduction, relevant mandibular function, disability, patient-prioritized functional goals, and safety considerations. Additional measures of psychosocial burden, sleep, headache, regional symptoms, awake bruxism or pain sensitivity should be selected when suggested by the clinical presentation (Supplementary Material Tables S1–S4). Outcome monitoring should similarly reflect the dominant phenotype and the goals agreed with the patient.

### 11.3. Interpretation over Time

Phenotypes should be regarded as dynamic working formulations. Their relative importance may change during treatment as pain decreases, movement improves, behavior changes or previously less relevant comorbidities become clinically prominent. Reassessment therefore serves not only to document outcome, but also to determine whether the original treatment hypothesis remains plausible and whether treatment targets, intensity or referral decisions should be modified.

## 12. Limitations of the Proposed Framework

Several limitations should be acknowledged. This framework is a clinical proposal and has not yet been validated as a treatment-selection algorithm. Its categories should be interpreted as pragmatic clinical categorization rather than definitive biological subtypes. The proposed phenotypes may overlap. A patient may simultaneously present with mobility limitation, awake bruxism, poor sleep, headache comorbidity and high distress. In such cases, the framework requires the clinician to identify the dominant treatment target rather than assign the patient to a single rigid category.

The evidence supporting conservative interventions in TMD is heterogeneous, and treatment effects may vary according to diagnosis, chronicity, comorbidity, pain mechanism, psychosocial burden and outcome selection. Some components of the framework, particularly ecological momentary intervention for awake bruxism, pain-mechanism-informed phenotyping and phenotype-specific treatment matching, require further prospective evaluation.

Standardized assessment is essential but does not eliminate clinical uncertainty. Palpation, joint play, movement assessment and motor-control interpretation may be influenced by examiner skill, patient sensitivity and contextual factors. Psychosocial screening tools identify risk and burden but do not provide a psychological diagnosis. Their use should support communication and referral, not labeling.

The proposed low-, moderate- and high-complexity categories are intended as pragmatic clinical strata. Specific cut-offs for escalation, treatment dose and referral thresholds have not yet been established. Finally, this framework was developed through narrative synthesis rather than formal consensus or a GRADE-based methodology. Future versions should be refined through Delphi consensus, feasibility testing, reliability studies and prospective validation.

## 13. Research Agenda

Future research should address several priorities. The first stage of framework development should involve an international Delphi study to establish multidisciplinary expert consensus on its domains, terminology, clinical applicability, and proposed decision-making pathway. The reliability of physiotherapy-specific phenotype allocation should be tested across clinicians with different training levels. Prospective cohort studies should evaluate whether proposed phenotypes predict treatment response, persistence of symptoms, recurrence or need for interdisciplinary care. Pragmatic randomized trials should compare phenotype-matched rehabilitation with usual conservative care.

Ecological momentary assessment and ecological momentary intervention strategies for awake bruxism should be tested for clinically relevant outcomes, including pain, function, disability, patient awareness and long-term adherence.

Future studies should evaluate the responsiveness of phenotype-specific outcomes, including mandibular range of motion, jaw function, headache burden, sleep quality, patient-specific function and psychosocial measures. Escalation criteria for low-, moderate- and high-complexity pathways should be developed and validated. Implementation studies should evaluate whether a minimum dataset and extended dataset can be feasibly integrated into routine physiotherapy practice. Finally, studies should assess whether this framework improves communication among physiotherapists, dentists, orofacial pain specialists, neurologists, sleep-medicine clinicians and psychologists.

## 14. Conclusions

DC/TMD, ICOP and ICHD-3 provide essential diagnostic foundations for TMD and orofacial pain, but physiotherapy management often requires an additional translational step. The proposed framework aims to bridge diagnosis and treatment by integrating screening for serious conditions, differential diagnosis, modifiable local contributors, pain mechanisms, movement impairments, behavioral perpetuating factors, regional comorbidities, sleep and prognostic indicators.

In this model, physiotherapy assessment is not a search for a single structural cause, but a process of clinical phenotyping that links diagnosis to individualized conservative care. Diagnostic anchoring identifies the condition; physiotherapy phenotyping identifies the dominant modifiable target, the appropriate intervention intensity and the outcomes that should be monitored.

Manual therapy, exercise, education, and behavioral strategies may be selected in relation to explicit treatment targets rather than applied as generic protocols. Joint sounds and structural findings should be interpreted cautiously, particularly when they are painless and functionally irrelevant. Awake bruxism should be conceptualized as a potentially relevant behavior in selected patients, not as a universal pathology. High disability, distress, widespread symptoms, poor sleep, frequent headache comorbidity and poor recovery indicators may indicate the need to consider broader stepped care and interdisciplinary collaboration.

This framework is intended to support clinical reasoning and generate testable hypotheses. Its value will depend on future research assessing feasibility, reliability, prognostic validity, clinical effectiveness and implementation in routine physiotherapy practice. Until then, it should be interpreted as an evidence-informed proposal for individualized physiotherapy management in patients with TMD.

## Figures and Tables

**Table 1:** Pain-mechanism-informed phenotyping in physiotherapy-oriented TMD care.

Proposed Pain-Mechanism Profile	Clinical Indicators	Potential Physiotherapy Implication
Predominantly nociceptive features	Familiar pain reproduction; load-related symptoms; localized pain; proportional response to jaw function or palpation	Focused local conservative care, load management, mobility or strengthening exercise, symptom-modulating adjuncts
Features raising suspicion of neuropathic pain	Electric-shock-like pain, burning pain, paresthesia, sensory deficit, allodynia in a neuroanatomical distribution, cranial nerve signs	Diagnostic caution, referral or co-management, avoid inappropriate repeated mechanical treatment
Nociplastic/high-sensitivity presentation	Disproportionate or widespread pain, generalized hypersensitivity, poor sleep, fatigue, high somatic burden, multiple comorbid pain complaints	Pain education, graded activity, interdisciplinary care, symptom-informed progression

**Table 2:** Stratified stepped care according to clinical complexity—These complexity strata and escalation considerations are hypothesis-generating clinical propositions, partly extrapolated from broader musculoskeletal stratified-care models, and have not yet been prospectively validated in TMD.

Complexity Level	Entry Criteria	Treatment Intensity	Escalation Criteria
Low complexity	Localized familiar pain; low disability; low distress; good sleep; limited comorbidity; clear modifiable mechanical or behavioral contributor	Brief focused care; education; reassurance; self-management; targeted exercise; short-term adjunctive manual therapy when clinically justified	No meaningful improvement after 4–6 weeks; unexpected worsening; emerging red flags or diagnostic uncertainty
Moderate complexity	Higher pain intensity; functional limitation; movement avoidance; ROM limitation; persistent awake bruxism; sleep complaints; relevant neck pain, migraine or tension-type headache comorbidity	Structured rehabilitation over several weeks; exercise progression; motor-control training; behavioral strategies; pain education; sleep-informed advice; monitoring of patient-specific outcomes	Persistent disability; poor response to appropriate care; increasing headache burden; relevant sleep or psychological concerns
High complexity	High disability; severe distress; widespread symptoms; high somatic symptom burden; poor sleep; frequent migraine or recurrent tension-type headache; multiple comorbid pain conditions; poor response to local treatment	Slower and broader pathway; pain education; graded activity; pacing; self-efficacy; functional restoration; interdisciplinary care	Medical, neurological, psychological, dental, sleep-medicine or pain-specialist referral according to dominant complexity drivers

## Data Availability

No new data were created or analyzed in this study. Data sharing is not applicable to this article.

## Supplementary Materials

The following supporting information can be downloaded at https://www.mdpi.com/article/10.3390/jpm16100529/s1: Table S1. Proposed physiotherapy assessment domains in TMD; Table S2. From physiotherapy assessment to treatment target; Table S3. Suggested outcome monitoring according to physiotherapy phenotype; Table S4. Clinical confidence in selected framework components.

## Abbreviations

The following abbreviations are used in this manuscript:

DC/TMD	Diagnostic Criteria for Temporomandibular Disorders
EMA	Ecological Momentary Assessment
EMI	Ecological Momentary Intervention
GAD-7	Generalized Anxiety Disorder 7-item scale
GCPS	Graded Chronic Pain Scale
GRADE	Grading of Recommendations Assessment, Development and Evaluation
IASP	International Association for the Study of Pain
ICHD-3	International Classification of Headache Disorders, 3rd edition
ICOP	International Classification of Orofacial Pain
JFLS	Jaw Functional Limitation Scale
OPPERA	Orofacial Pain Prospective Evaluation and Risk Assessment
PHQ-4	Patient Health Questionnaire-4
PHQ-9	Patient Health Questionnaire-9
PHQ-15	Patient Health Questionnaire-15
RDC/TMD	Research Diagnostic Criteria for Temporomandibular Disorders
ROM	Range of Motion
TMD	Temporomandibular Disorder
