# Supplementary material for: A Physical Therapy Clinical Framework for Temporomandibular Disorders: Integrating DC/TMD, ICOP and Multidimensional Patient Phenotyping
Source: J Pers Med. 2026 Oct 14;16(10):529. doi: 10.3390/jpm16100529 (PMC13706627; doi:10.3390/jpm16100529)
Supplement: Supplementary file 1 [file jpm-16-00529-s001.zip › jpm-4500809-supplementary.pdf]

Supplementary Materials

Domain	Main clinical question	Suggested assessment components	Clinical interpretation
Diagnostic anchoring	What is the most appropriate diagnostic category and are there competing diagnoses?	DC/TMD diagnosis, ICOP/ICHD-3-informed differential diagnosis	Establishes diagnostic boundaries and avoids treating non-TMD conditions as TMD
Screening for serious conditions	Can this patient be safely managed within physiotherapy scope?	Red flags, severe pathology screening, non-TMD differential diagnosis, referral criteria	Identifies need for referral before treatment
Screening for specific conditions	Are there conditions requiring co-management?	Rheumatological features, clinically relevant sleep bruxism, sleep-disordered breathing, complex headache presentations	Supports interdisciplinary management
Peripheral nociceptive contributors	Which local findings reproduce familiar pain or modify symptoms?	Standardized palpation, joint loading, joint play, symptom modification tests	Identifies potentially modifiable peripheral contributors
Pain-mechanism profile	Does the presentation show predominantly nociceptive features, features raising suspicion of neuropathic pain, or a high-sensitivity presentation with possible nociplastic features?	Symptom quality, distribution, proportionality, sensory features, widespread symptoms, sleep, somatic burden	May inform treatment emphasis, pacing, referral and interdisciplinary management
Function and motor control	How does the patient move and function?	Maximum mouth opening, lateral/protrusive movement, deviation, coordination, chewing and yawning tolerance	May inform mobility, coordination, graded exposure or functional exercise
Behavioral load	Are daily behaviors maintaining overload or vigilance?	Awake bruxism history, EMA when available, rest-position awareness, stress-related jaw activity	May inform behavioral awareness, EMI, relaxation and load management
Regional comorbidities	Do neck pain, migraine or tension-type headache modify prognosis or treatment?	Neck-related symptoms, headache frequency, headache disability, symptom modification, medication use	May inform broader regional reasoning, co-management and monitoring
Sleep-pain burden	Is sleep disturbance reducing recovery or suggesting referral?	Sleep quality, morning fatigue, morning jaw pain, snoring, witnessed apneas, daytime sleepiness	May inform sleep-informed advice and referral when indicated
Prognosis and complexity	How intensive and interdisciplinary should care be?	GCPS, PHQ-4/PHQ-9/GAD-7, PHQ-15, JFLS, pain drawing, comorbid symptoms	May inform stepped care, monitoring intensity and referral

Table S1. Proposed physiotherapy assessment domains in TMD

Dominant phenotype	Typical findings	Primary treatment target	Intervention emphasis	Referral considerations
Referral	Red flags, suspected severe pathology, condition outside physiotherapy scope	Ensure appropriate diagnosis and safety	No direct physiotherapy treatment before referral	Medical, dental, neurological or emergency referral according to presentation
Specific-condition	Suspected rheumatological disorder, clinically relevant sleep bruxism, suspected sleep-disordered breathing or complex headache presentation	Integrate physiotherapy within broader care	Education and conservative management only when appropriate	Interdisciplinary management
Mobility-deficit	Restricted mouth opening, limited lateral/protrusive movement, locking history, functional range limitation	Improve movement tolerance and functional range	Graded mobility exercise, selected joint mobilization, functional exposure	Dental/orofacial pain referral if locking, trauma, progressive limitation or diagnostic uncertainty
Nociceptive (myofascial/arthralgia)	Familiar pain reproduced by masseter/temporalis palpation, localized symptoms, low disability, limited comorbidity	Reduce local nociceptive input and restore comfortable function	Education, self-management, jaw relaxation, load management, soft-tissue techniques as adjunct, progressive exercise	Usually not required initially
Motor-control	Deviation, jerky opening, guarding, poor coordination, fear of movement	Restore confident and controlled mandibular movement	Controlled opening, visual feedback, graded exposure, task-specific exercise	Consider psychological input if fear and avoidance dominate
Behavioral-load	Frequent jaw bracing, tooth contact or clenching during wakefulness; muscle fatigue or pain	Reduce sustained non-functional masticatory muscle activity	Education, EMA/EMI, rest-position training, stress-context mapping	Consider psychological support when behavior is linked to high distress
Regional-comorbidity	Neck pain, migraine, tension-type headache, cranio-cervical symptom overlap	Integrate jaw management with regional symptom behavior	Headache/neck screening, regional load management, outcome monitoring, co-management when needed	Medical, neurological or orofacial pain collaboration when headache is complex or poorly controlled

Dominant phenotype	Typical findings	Primary treatment target	Intervention emphasis	Referral considerations
Sleep-pain burden phenotype	Poor sleep quality, non-restorative sleep, fatigue, morning symptoms, suspected sleep bruxism or sleep-disordered breathing	Improve recovery capacity and identify sleep-related referral needs	Sleep-pain education, pacing, evening load reduction, basic sleep hygiene within scope	Sleep-medicine referral when sleep-disordered breathing or complex insomnia is suspected
High-sensitivity presentation with possible nociplastic features	Disproportionate or widespread pain, generalized sensitivity, poor sleep, high somatic symptom burden	Improve self-efficacy, function and global pain management	Pain education, graded activity, pacing, gentle exercise, interdisciplinary care	Strongly consider multidisciplinary pain, psychology, medical or dental referral
High-impact psychosocial	High disability, severe distress, fear, low self-efficacy, poor response to local treatment	Reduce threat, restore function and coordinate care	Education, graded exposure, functional goals, interdisciplinary management	Psychological, medical or multidisciplinary pain referral

Table S2. From physiotherapy assessment to treatment target - The proposed phenotype–target relationships are hypothesis-generating clinical propositions and should not be interpreted as validated treatment-selection rules.

Phenotype	Minimum outcome monitoring
Mobility-deficit	Maximum mouth opening, lateral/protrusive movement, chewing and yawning tolerance
Myofascial or joint nociceptive	Familiar pain intensity, pain during chewing, local pressure tolerance, symptom response to load management
Motor-control	Movement smoothness, deviation/deflection behavior, movement confidence, task performance
Behavioral-load	Awake bruxism frequency, perceived jaw tension, fatigue episodes, EMA-derived behavior patterns when available
Regional-comorbidity	Headache days, headache disability, neck-related symptoms, medication use, symptom modification
Sleep-pain burden	Sleep quality, fatigue, morning symptoms, snoring/daytime sleepiness screening when indicated
High-sensitivity presentation with possible nociplastic features	GCPS, PHQ-4, PHQ-15, pain distribution, patient-specific function
High-impact psychosocial	Disability, self-efficacy, fear/avoidance, distress screening, patient-specific functional goals

Table S3. Suggested outcome monitoring according to physiotherapy phenotype

Component	Evidence basis	Clinical confidence	Comment
Direct-access screening and referral	Clinical safety principles and guideline-based care pathways	High	Essential before conservative management
DC/TMD-based diagnosis and familiar pain	International diagnostic standards	High	Strong foundation for diagnosis and communication
ICOP/ICHD-3-informed differential diagnosis	International classification systems	High	Essential to avoid diagnostic tunnel vision
Education and conservative self-management	Guidelines and clinical consensus	High	Should be a foundation for most patients
Supervised therapeutic exercise	Systematic reviews and guidelines	Moderate to high	Effects are generally favorable but protocols vary
Manual therapy as adjunct	Systematic reviews and guidelines	Moderate	Best framed as selected adjunct to active care
Pain-mechanism-informed phenotyping	Musculoskeletal pain literature and IASP nociplastic pain criteria	Low to moderate	Clinically useful but requires validation in TMD physiotherapy
EMA for awake bruxism	Consensus and growing observational literature	Moderate	Useful for assessment and awareness; treatment effects require further study
EMI for awake bruxism	Emerging and indirect evidence	Moderate	Promising but not yet a validated stand-alone intervention

Component	Evidence basis	Clinical confidence	Comment
Stratified stepped care	Evidence from other musculoskeletal fields and clinical reasoning	Low to moderate	Requires TMD-specific feasibility and outcome testing
Phenotype-based treatment matching	Clinical reasoning supported by multidimensional pain literature	Low to moderate	Central proposal of this paper; requires prospective validation
Outcome-based monitoring	Clinical measurement principles and guideline-based care	Moderate	Necessary to test response and guide escalation

Table S4. Clinical confidence in selected framework components
